# Acute hemodynamic effects of L-arginine, arginine nitrate, and arginine peptide on exercise-induced vasodilation and blood flow in healthy men

**DOI:** 10.1186/1550-2783-12-S1-P10

**Published:** 2015-09-21

**Authors:** Paul H Falcone, Jordan M Joy, Roxanne M Vogel, Matt M Mosman, Aaron C Tribby, Chad M Hughes, Jonathan D Griffin, Schyler B Tabor, Dylan J LeFever, Stephen B McCaughey, Michael P Kim, Jordan R Moon

**Affiliations:** 1 MusclePharm Sports Science Institute, Denver, CO, USA; 2 Department of Human Performance, Concordia University Chicago, River Forest, IL, USA; 3 Department of Movement Science, Grand Valley State University, Allendale, MI, USA; 4 Department of Biomedical Engineering, Widener University, Chester, PA, USA; 5 The Hospitality College, Johnson and Wales University, Denver, CO, USA; 6 Department of Human Performance and Sport, Metropolitan State University, Denver, CO, USA; 7 Department of Sports Exercise Science, United States Sports Academy, Daphne, AL, USA

## Background

Increasing blood flow to skeletal muscle during exercise may benefit both recreational and elite athletes. Raw arginine (RA) is a commonly used supplement for increasing blood flow via nitric oxide production. Arginine has been also been bound to a whey peptide (AP) and to nitrate (AN) to increase bioavailability. The purpose of the present study was to determine the acute hemodynamic effects of RA, AP, AN, and placebo (PLA) following resistance exercise in healthy, recreationally-active men at doses commonly used in the marketplace.

## Methods

In a double-blind, crossover, placebo-controlled design, 11 recreationally-active males (28.2 ± 5.0y, 182.4 ± 5.7cm, 87.1 ± 10.3kg) consumed either 1.87 g of RA, 3.07 g of AP (arginine content 1.87g), 2.55g of AN (arginine content 1.87g), or a flavor-matched, visually identical placebo (PLA), and performed 3 sets of 15 arm curls at 30 and 120 minutes post-supplementation. Vessel diameter of the brachial artery (VD) and blood flow volume (BFV) were measured via Doppler ultrasound at 0, 3, and 6 minutes post-exercise, corresponding to 30 (30P), 33 (33P), 36 (36P), 120 (120P), 123 (123P), and 126 (126P) minutes post-supplementation. Measurements were compared with active control (no treatment, exercise) values. Raw data were analyzed for all group, time, and group × time interactions using 2-way repeated-measures ANOVA. Percent change values were analyzed using dependent t-tests. Alpha was set at p < 0.05.

## Results

A significant (p < 0.05) group × time interaction was observed for RA compared to PLA, and post hoc analyses revealed that RA increased VD versus PLA at 30P (RA: 0.56 ± 0.17; PLA: 0.55 ± 0.17cm) compared to control. Significantly greater percent change values were observed for VD when comparing RA and PLA at 30P versus active (RA: 7.87 ± 4.09; PLA: 3.90 ± 3.75cm). Significantly greater percent change values were observed for BFV when comparing AP and PLA at 33P, AP and RA at 33P, AP and PLA at 36P, and AP and AN at 123P and 126P versus active baselines ([AP 33P: 25.7 ± 39.1; 36P: 22.0 ± 41.6; 123P: 21.5 ± 47.6; 126P: 3.02 ± 31.7mL/min], [PLA 33P: 3.27 ± 30.6; 36P: 5.71 ± 33.6mL/min], [RA 33P: -0.71 ± 34.5mL/min], [AN 123P: -2.58 ± 29.6; 126P: -21.8 ± 27.6mL/min]).

## Conclusions

Though raw arginine may significantly increase vessel diameter compared to placebo at 30 minutes post-exercise, arginine peptide induced significantly higher percent change values for blood flow volume compared to raw arginine, placebo and arginine nitrate at specific time points, and therefore may be the best option for increased blood flow.

